# Cholera Infantum

**Published:** 1882-08

**Authors:** 


### Editorial.

### "CHOLERA INFANTUM."

The advent of warm weather is invariably accompanied by a frequent recurrence of the name of this summer scourge of infancy and early childhood upon our city bills of mortality, and the evidence furnished by these periodical reports, tells a tale of weary watching and anxious suspense, succeeded, alas, too often by blasted hopes and domestic desolation, in many elegant and luxurious homes all over this favored land.

It would not be appropriate, in this place, to enter into a full consideration of the diseased conditions embraced within the scope of this elastic and pliable term; but we feel justified in inviting attention, very briefly and of necessity very imperfectly, to some of the more important features of this subject, as they appear to us.

Divested of all complications and sheared of all subsequent manifestations, the disorder under review may be reduced at the outset, in the vast majority of cases, at least, to a simple diarrhœa—a simple catarrhal condition of the gastro-intestinal tract—occasioned in most instances, according to our belief, by the ingesta—by improper food, that is, food which, for the child, is indigestible and which consequently remains undigested—a source of constant irritation—a cause of grave disease.

It should be borne in mind that the alimentary canal and the other digestive organs of infants are in a rudimentary or imperfectly developed state, hence the absurdity and the actual danger of expecting them to appropriate, in the first year or two of life, even some of the blandest articles of diet habitually employed without detriment by adults.

That many substances, not infrequently used as food for infants are not only totally unfit for purposes of nutrition in them, but by their very presence set up a disordered action and are positively injurious, cannot be denied. This kind of ingesta not only fails to supply needed nourishment, but acting as a foreign body may excite and maintain a severe irritation of the stomach and bowels; and thus is taken the first step in the series of phenomena constituting cholera infantum, so-called.

Undoubtedly children are often predisposed to this class of disorders by their surroundings and circumstances—by conditions that serve to depress the nervous energy and to diminish the nervous power. But in many, possibly in most, of these cases this diminution of nervous force may be successfully met by lessening the tax daily put upon the digestive organs and by lightening the strain daily placed upon the digestive processes; and thus a direct exciting cause may be removed and a remote and far-reaching cause—starvation, and still further reduction of the nervous force—averted.

If the character and quality of the food suitable for young children is of such vital importance it surely becomes us to consider the whole question calmly, thoroughly and without prejudice.

In seeking an appropriate, a physiological food adapted to the requirements of infantile life, we certainly can find no safer, no surer guide than nature.

Following the lead of this pilot, we discover that milk—mother's milk—is provided by nature for the sustenance of human offspring during the earlier periods of its existence, and further that it is the food best suited to its necessities and to its development—possessing all the elements necessary to sustain and nourish the body.

In those unfortunate cases where this form of nourishment, from choice or necessity, is withheld, the plain line of duty lies in the direction of procuring an acceptable substitute. This substitute will not be found in any farinaceous preparation or in any of the numerous "infants' foods" that flood the market and curse the country, urged upon an innocent and uninformed public by enterprising manufacturers and interested individuals, aided, unhappily, not infrequently, by the implied or expressed indorsement of reputable medical practitioners. But it can be found in cows milk. Cows milk properly selected and properly prepared will meet the demand, as nothing short of human milk will.

A great deal, it should be distinctly understood, everything, indeed, depends upon the fidelity exhibited in the selection of milk, and in its preparation; but it is a matter of such great importance as to call for the most studious and intelligent attention to every detail of its procurement, preparation and administration. Of course, no invariable rule can be laid down to govern the extent of dilution to which the milk should be subjected; this, and other details of its preparation depend upon the age, the physical condition and the degree of development of the child, and should be determined by a competent adviser in each individual case.

If, after the exercise of due care and caution and the oberservance of rigid dietary and hygienic rules, a tendency to diarrhœa should be noted, it is not, under these circumstances, good practice to rely upon opiates and astringents to relieve the sign of the derangement, but the catarrhal symptoms should be accepted as evidence of an enfeebled digestion. The milk should then be diluted beyond the standard adopted in health, and thereby rendered more easy of digestion, and aids to the digestion should be given. The medication, if any should be required, should be directed, not to the arrest of a symptom of the disorder merely, but toward removal of the cause. The object should be not to obscure the signal of danger displayed as a warning by nature, or to arrest the effort of nature to lay off the unnatural burden, but toward rendering the food easier of absorption and assimilation and toward stimulating the digestive system to increased functional activity.

We do not hesitate to express the conviction that faithful adherence to the principles herein declared and to the plan herein imperfectly sketched will realize great advantages over the too prevalent practices of the present day, and that the use, as a constant diet, of a physiological food, or, in its unavoidable absence, of the nearest approach thereto that art can supply by a proper and exact preparation of the milk of a well chosen cow, so that its several constituent elements shall be made to conform as nearly as possible to the proportions found in human milk, and skillfully adjusted to the requirements of the individual child, will, in a single year, turn aside much sorrow and reserve for future usefulness the lives of many little victims of a preventable but fearfully fatal disease.

